# Newborn Screening for SCID and Other Severe Primary Immunodeficiency in the Polish-German Transborder Area: Experience From the First 14 Months of Collaboration

**DOI:** 10.3389/fimmu.2020.01948

**Published:** 2020-10-16

**Authors:** Maria Giżewska, Katarzyna Durda, Theresa Winter, Iwona Ostrowska, Mariusz Ołtarzewski, Jeannette Klein, Oliver Blankenstein, Hanna Romanowska, Elżbieta Krzywińska-Zdeb, Michał Filip Patalan, Elżbieta Bartkowiak, Natalia Szczerba, Stefan Seiberling, Bożena Birkenfeld, Matthias Nauck, Horst von Bernuth, Christian Meisel, Ewa Anna Bernatowska, Mieczysław Walczak, Małgorzata Pac

**Affiliations:** ^1^Department of Pediatrics, Endocrinology, Diabetology, Metabolic Diseases and Cardiology, Pomeranian Medical University, Szczecin, Poland; ^2^Independent Public Clinical Hospital nr 1 PUM, Szczecin, Poland; ^3^Institute of Clinical Chemistry and Laboratory Medicine, University Medicine Greifswald, Greifswald, Germany; ^4^Integrated Research Biobank (IRB), University Medicine Greifswald, Greifswald, Germany; ^5^Department of Screening and Metabolic Diagnostics, Institute of Mother and Child, Warsaw, Poland; ^6^Newbornscreening Laboratory, Charité Universitaetsmedizin, Berlin, Germany; ^7^Research Support Center, University of Greifswald, Greifswald, Germany; ^8^Department of Nuclear Medicine, Pomeranian Medical University, Szczecin, Poland; ^9^DZHK (German Centre for Cardiovascular Research), Partner Site Greifswald, University Medicine Greifswald, Greifswald, Germany; ^10^Department of Pediatric Pulmonology, Immunology and Intensive Care Medicine, Charité - Universitätsmedizin Berlin, Berlin, Germany; ^11^Labor Berlin - Charité Vivantes Services GmbH, Berlin, Germany; ^12^BIH Center for Regenerative Therapies, Charité - Universitätsmedizin Berlin, Berlin, Germany; ^13^Institute of Medical Immunology, Charité - Universitätsmedizin Berlin, Berlin, Germany; ^14^Department of Immunology, The Children's Memorial Health Institute, Warsaw, Poland

**Keywords:** newborn screening, SCID, TREC, KREC, RareScreen, PID, NGS

## Abstract

In 2017, in the Polish-German transborder area of West Pomerania, Mecklenburg-Western Pomerania, and Brandenburg, in collaboration with two centers in Warsaw, a partnership in the field of newborn screening (NBS) for severe primary immunodeficiency diseases (PID), mainly severe combined immunodeficiency (SCID), was initiated. SCID, but also some other severe PID, is a group of disorders characterized by the absence of T and/or B and NK cells. Affected infants are susceptible to life-threatening infections, but early detection gives a chance for effective treatment. The prevalence of SCID in the Polish and German populations is unknown but can be comparable to other countries (1:50,000–100,000). SCID NBS tests are based on real-time polymerase chain reaction (qPCR) and the measurement of a number of T cell receptor excision circles (TREC), kappa-deleting recombination excision circles (KREC), and beta-actin (ACTB) as a quality marker of DNA. This method can also be effective in NBS for other severe PID with T- and/or B-cell lymphopenia, including combined immunodeficiency (CID) or agammaglobulinemia. During the 14 months of collaboration, 44,287 newborns were screened according to the ImmunoIVD protocol. Within 65 positive samples, seven were classified to immediate recall and 58 requested a second sample. Examination of the 58 second samples resulted in recalling one newborn. Confirmatory tests included immunophenotyping of lymphocyte subsets with extension to TCR repertoire, lymphoproliferation tests, radiosensitivity tests, maternal engraftment assays, and molecular tests. Final diagnosis included: one case of T-B^low^NK+ SCID, one case of atypical T^low^ B^low^NK+ CID, one case of autosomal recessive agammaglobulinemia, and one case of Nijmegen breakage syndrome. Among four other positive results, three infants presented with T- and/or B-cell lymphopenia due to either the mother's immunosuppression, prematurity, or unknown reasons, which resolved or almost normalized in the first months of life. One newborn was classified as truly false positive. The overall positive predictive value (PPV) for the diagnosis of severe PID was 50.0%. This is the first population screening study that allowed identification of newborns with T and/or B immunodeficiency in Central and Eastern Europe.

## Introduction

Newborn screening (NBS) tests enable identification of infants with life-threating disorders, which require early intervention shortly after birth.

NBS was initially implemented in the early 1960's in the United States for the detection and treatment of phenylketonuria (1). Within the next years, this test was introduced in many other countries worldwide. Many rare genetic diseases, including inborn errors of metabolism and endocrine disorders, were successfully implemented to newborn screening programs later on. Different analytical techniques were used. These included bacterial inhibition tests, radioimmunoassays, immunoassays with colorimetric, fluorometric, or luminometric measurements and, starting from the late 1990's, tandem mass spectrometry followed soon after by DNA-based technologies (2, 3). In 2005, McGhee et al. and Chan and Puck described the first-time application of the DNA–based assay (T-cell receptor excision circles—TREC) colorimetric NBS for severe combined immunodeficiency (SCID) and other forms of T-cell lymphopenia (4, 5). Some years later, KREC (kappa–deleting recombination circles) was proposed for a combined TREC—KREC screening approach for severe forms of T- and/or B-cells deficiencies, such as SCID, late-onset adenosine deaminase deficient SCID (ADA-SCID), combined immunodeficiency (CID), or different forms of agammaglobulinemia (XLA) (6, 7). Additionally, in ADA-SCID the purine metabolites and adenosine deaminase activity can be determined using tandem mass spectrometry (8, 9).

Primary immunodeficiency diseases (PID) are a heterogenous group of inborn errors of immunity affecting ~1 in 10,000 to 1 in 50,000 births (10). Currently, over 400 different genetic mutations and diseases are recognized, yet the collective prevalence is likely to be higher (11–13). Patients with PID are classified into one out of 10 groups, such as predominant antibody deficiency, cellular deficiency, combined immunodeficiency, and others (13). The leading symptom of the majority of them is a predisposition to life-threating infections.

SCID is the most severe form of PID and represents a group of rare inborn defects of the immunity with either known (about 20) or unknown gene defects. The most common feature is the lack or very diminished number of T-cells, accompanied by absent or non-functional B lymphocytes and NK cells (13, 14). Affected individuals appear to be healthy at birth. They start to present infections and failure to thrive at the age of ~3–6 months. Severe and recurrent infections of bacterial, viral, fungal, and opportunistic origin, as well as secondary infections induced by life vaccines, are life-threatening with fatal outcomes within the first 1–2 years of life if untreated (15). Since the late 1960's, many advances have been made in the field of treatment, including hematopoietic stem cell transplantation (HSCT), enzyme therapy, or gene therapy for different genetic forms of SCID (16, 17).

As a condition which is potentially curable if recognized and treated early, SCID and other severe PID meet Wilson's and Jungner's criteria for NBS (18, 19). TREC assay for NBS dedicated to the early diagnosis of SCID was implemented for the first time in the United States in 2008 (20). Several studies performed since that time revealed a SCID incidence of 1: 58,000 live births (21). Now it is screened throughout the United States (as one out of 35 primary screened disorders included in the recommended uniform screening panel), as well as in some countries in Europe and other continents, either reimbursed by governments or by scientific/pilot studies (2, 22).

Outside the United States, routine TREC screening is currently performed in Israel, New Zealand, Norway, Taiwan, several provinces in Canada, Switzerland, Iceland, Sweden, Italy (Tuscany), Spain (Catalonia), and in some regions in Australia (15). The overall implementation status EU-wide is very diverse. Considerations are still going on in various European countries. Pilot projects are currently underway or have been announced in France (23), Spain (24, 25), Norway, and the Netherlands (26).

In Germany, prior to the nationwide implementation of SCID NBS, only a few screening laboratories had experience with this procedure, such as laboratories in Munich (head: B. Olgemoeller), Heidelberg (head: G.F. Hoffmann), Hannover (head: N. Janzen), Leipzig (head: J. Thiery), and Greifswald (head: M. Nauck) (27). The whole process to include SCID in newborn screening began in 2012 with a local pilot trial in Leipzig. From 2014 to 2016, another pilot project was carried out in Heidelberg. Finally, in August 2019 the SCID NBS was implemented nationwide (TREC only) (28). We present the results of the first 14 months of the trans-border cooperation in the field of NBS for SCID and other severe PID in the area of West Pomerania, Poland, and Mecklenburg-Western Pomerania and Brandenburg in Germany.

## Materials and Methods

The Polish-German transborder cooperation in the field of NBS for SCID and other severe PID was possible thanks to the Cooperation Program Interreg V A Mecklenburg-Vorpommern/Brandenburg/Poland and the project entitled “Innovative Polish-German cross-border program for early diagnosis and treatment of rare diseases in newborns–RareScreen” (INT10). The project was intended to expand the NBS panel for inborn errors of metabolism and other rare diseases including SCID. It was initiated on 01.05.2017 and will last till 30.10.2020.

The “RareScreen” project includes newborns born in the Euro-Pomerania region which covers West-Pomerania in Poland and Mecklenburg-Western Pomerania and part of Brandenburg in Germany. The population of the above-mentioned geographical regions in 2016 was as follows: 724,161 inhabitants in Mecklenburg-Vorpommern, 300,243 in Brandenburg (1,024,404 in total), and 1,708,174 in West Pomerania, Poland (29).

**Figure 1:**
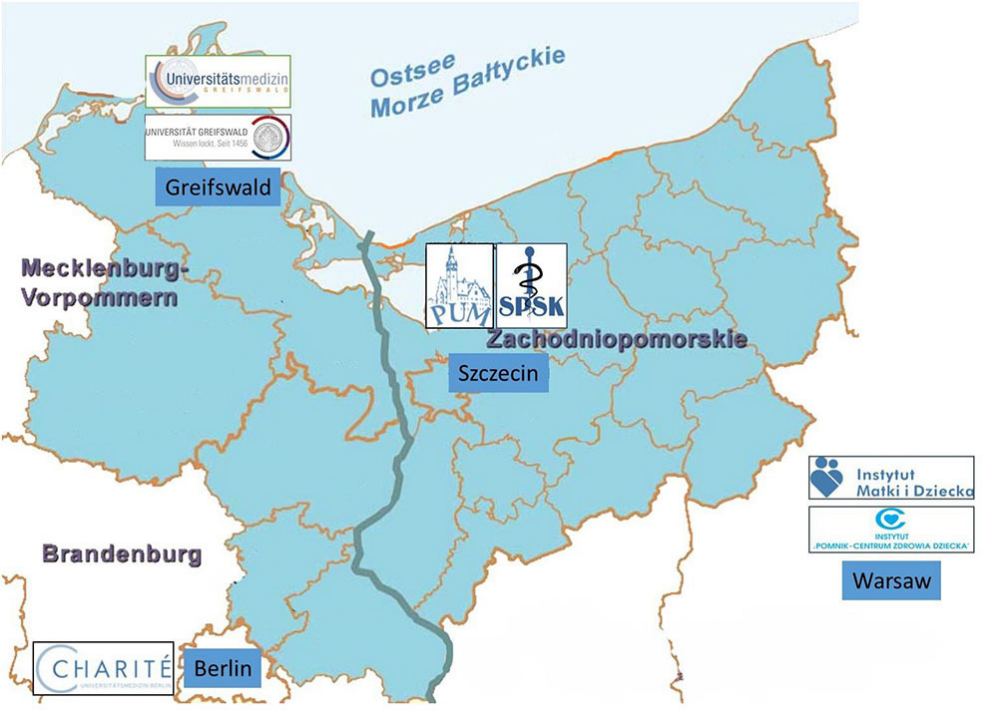
The region covered by the Rare-Screen Project according to the Cooperation Program Interreg V A Mecklenburg-Vorpommern/ Brandenburg/ Poland with the participating German and Polish partner sites from Greifswald, Berlin (D), Szczecin, and Warsaw (PL).

Three screening laboratories are involved in the project: Newborn Screening Laboratory Independent Public Clinical Hospital nr 1 PUM, Szczecin, Poland; Newborn Screening Laboratory, University Medicine Greifswald, Germany; and Newborn Screening Laboratory Charité University Medicine Berlin, Germany. The four other project partners are: University Medicine Greifswald, Germany; Pomeranian Medical University Szczecin, Poland; Institute of Mother and Child, Warsaw, Poland; and the Children's Memorial Health Institute, Warsaw, Poland (Figure 1).

The results presented here are based on samples which were collected between October 24, 2018 and December 31, 2019. The sample collection is currently still ongoing and will last until October 2020.

For all newborns, the TREC and KREC NBS assay was performed in the NBS Laboratory in the Independent Public Clinical Hospital nr 1 PUM Szczecin (West Pomerania, Poland).

The organization of efficient transborder transport of dry blood spots samples (DBS) was fundamental to the cooperation between the NBS laboratories. The DBS from Berlin to Greifswald were sent via regular post. Subsequently, DBS from both German centers were delivered via courier from Greifswald to Szczecin NBS Laboratory. The transport between Greifswald and Szczecin took place four times a week and the samples were transported and stored at 4°C until use. The samples reached Szczecin usually within 1–7 days after their arrival in the local laboratories. The results were electronically transferred from Szczecin to Greifswald and Berlin within two working days. Waiting time for repetition from the first screening card of German newborns took from 2 to 10 days. Repetitions of the test from the first screening card of Polish newborns were performed in the next protocol which took place on the next working day. If any further action was needed, e.g., the necessity of taking a second screening card or admission of a newborn to the hospital for confirmatory tests, the procedure took place according to the protocols of each center.

Information brochures about the “RareScreen” program for parents and health care providers were distributed. Brochures included basic information about SCID, the goals and necessity of implementing this study, the advantages of participating in the project, and data protection. Additionally, informative meetings were organized for midwives, pediatricians, and other parties involved in the study. The aim of medical staff training and development of the brochures was to educate parents so that they could sign their informed consent to participate in the study.

No additional blood collection was needed from the newborns participating in the project apart from the blood usually taken for regular NBS. Heel prick blood samples were collected on filter paper (Whatman 903 for newborns from Poland and PerkinElmer 226 Ahlstrom for newborns from Germany) between the third and fifth day post-partum as part of the national NBS programs in Poland and Germany.

IT software (NeoBase) was adapted to transfer necessary data between centers involved in the project.

Demographic data, such as sex, birth date, data of sample collection, birth weight (BW), and gestational age (GA) of the newborns, was collected in the database (NeoBase). According to WHO definition, newborns were allocated to groups according to GA as follow: ≥38 weeks—born at term; ≥32–37 weeks—moderate preterm, ≥28–32 weeks—very preterm; <28 weeks—extremely preterm (30). Additionally, in the NeoBase other essential information about the newborns regarding their clinical condition, medicines taken (antibiotics, steroids), and blood transfusions as well as the mothers' history and treatment during pregnancy, was registered.

The NBS tests were performed using a commercial kit—SPOT-it ™ TK (ImmunoIVD, Sweden). The screening laboratory in Szczecin was adapted and equipped with instruments for the PCR test method. Staff members were trained by the manufacturer of the TREC and KREC assay.

The 3.2 mm DBS were punched directly into 96-well-filter plate (ImmunoIVD) using a Wallac DBS puncher (Perkin Elmer). After DBS extraction and DNA elution, the qPCR reaction was performed (QuandStudio5, ThermoScience). The number of copies/μL for TREC, KREC, and beta-actin (ACTB) were calculated using the standard curves method. The amount of ACTB copy numbers indicates the efficacy of DNA extraction from DBS samples. Plates include three quality control punches with defined T- and B-cell ranges: T-cell depleted, B-cell depleted, and T-and B-cell depleted, as well as one blank control (DBS not soaked in blood) (QCs were provided also by ImmunoIVD).

### Definition and Interpretation of the Results

The TREC and KREC assay is intended to screen newborns with the most severe forms of T- and B-cell lymphopenia (14, 31). According to the manufacturer's instructions, the cut off values are 6 copies/μL for TREC and 4 copies/μL for KREC.

In the case of abnormal results (TREC <6 copies/μL and/or KREC <4 copies/μL) or inconclusive results (ACTB <1,000 copies/μL), a sample was repeated (re-tested) in duplicate from the first DBS. In the case of a positive NBS result, the following procedure depended on the values obtained from the first screening card (3 punches). When the value of TREC was in range of 1–4 and/or KREC 1–6 copies/μL, parents or medical staff (if the child was still at the hospital) were informed by letter or by phone call about the necessity of taking the second blood sample. Numbers of TREC and/or KREC <1 copies/μL in the re-tested first DBS (urgent-positive) resulted in immediate recall of the newborn and admission to the Department of Pediatrics, Endocrinology, Diabetology, Metabolic Diseases, and Cardiology in Szczecin and/or the Department of Immunology, The Children's Memorial Health Institute, Warsaw, Poland or to the Department of Pediatric Pneumology and Immunology, Charité Universitaetsmedizin Berlin, Germany for a confirmatory diagnosis. The aim of our program was to detect severe forms of PID, SCID in particular (defined as T-cells <300 cells/μL), as well as to identify other severe forms of T- and/or B-cell lymphopenia, including CID, agammaglobulinemia, and secondary immunodeficiencies (32). The confirmatory diagnostics procedures included, beside routine pediatric examination and basic laboratory tests, detailed immunocytometry assay (T cells [CD3/CD4/CD8, T cell naivety [CD45RA and CD45RO], B cells [CD19], NK cells [CD3/CD16/CD56], recent thymic emigrants (RTE) [CD4/CD31/CD45RA], α/ß and γ/δ T cells [CD3/α/ß TCR/γ/δ TCR], lymphocyte proliferation tests (to PHA, anti-CD3, and Pansorbin), humoral immunity adjustment (immunoglobulin levels), cytogenetic tests (karyotype), molecular tests (New Generation Sequencing or single gene sequencing by Sanger), and, if available and needed, radiosensitivity tests, analysis of the TCR Vbeta repertoire, ADA and PNP enzyme activity levels, and anthropometry.

**Figure 2:**
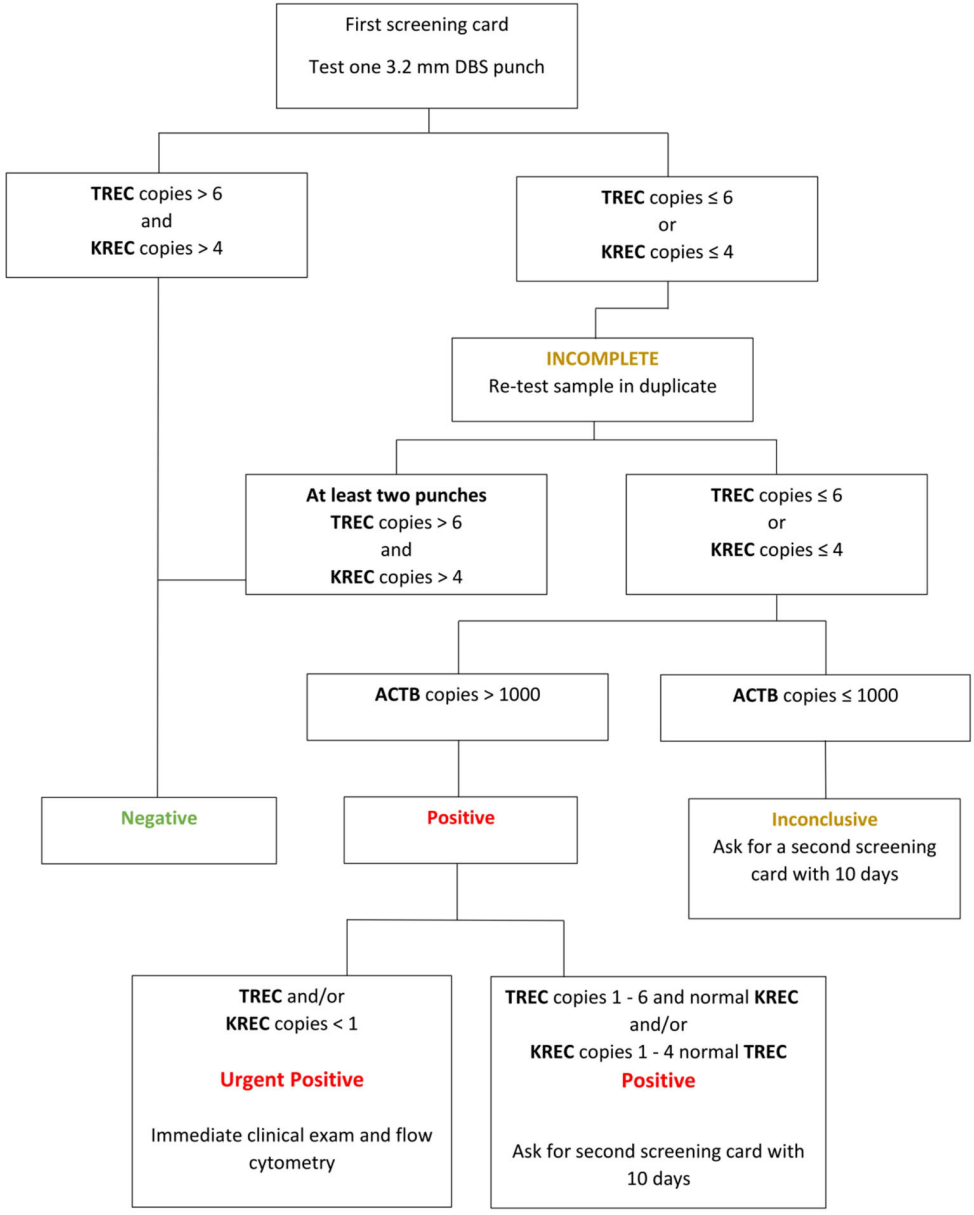
Interpretation of the SCID NBS results (TREC and KREC copies) according to ImmunoIVD.

False-positive results were defined when values for TRECs or KRECs in NBS were over the established cut-offs in absence of SCID or other PID in the confirmatory diagnosis (Figure 2).

Statistical analyses were performed using Python with the SciPy package. Given the beta distribution of the data, the Mann–Whitney test and the Kruskal–Wallis test were used to compare continuous variables between the groups. Differences were considered statistically significant when the *p*-value was <0.05.

## Results

A total of 44,287 newborn samples were prospectively collected from two centers in Germany (Greifswald−11,114; Berlin−15,428) and one in Poland (Szczecin−17,745). In this group of newborns, 22,804 (51.5%) were males and 21 434 (48.4%) were females. Information regarding the sex was incomplete in 49 (0.1%) cases and for 138 newborns (0.3%) GA was not provided. 36 634 (82.7%) newborns were born at term and 7,515 were preterm (17.0%). In a group of preterm newborns, there were 6,646 (15.0%) moderate preterm born at ≤ 32–37 weeks of gestation, 621 (1.4%) very preterm born between ≤ 28–31 weeks, and 248 (0.56%) extremely preterm children born <28 weeks. The median value for GA was 39 weeks (min. 22, max. 42 weeks). The median value for birth weight for the whole group was 3,395 g (min. 450 g, max. 5,504 g) (Table 1).

**Table 1:** Demographic data and median values of TREC and KREC copies/μL in the study population.

	**Sample size**	**TRECs, copies/μL, median (SD; range)**	**KRECs, copies/μL, median (SD; range)**
All newborns	44,287	86 (54.8; 0–830)	42 (33.0; 0–734)
**Sex**			
Male^*^	22,804 (51.49%)	81 (52.0; 0–830)	40 (32.2; 0–734)
Female^*^	21,434 (48.40%)	92 (56.1; 0–758)	44 (33.7; 0–600)
Unknown sex	49 (0.11%)	94	48
Term ≥ 38 weeks; *n* (%)	36,634 (82.71%)	88 (54.8; 0–830)	42 (31.8; 0–400)
Moderate preterm, ≥32–37 weeks; *n* (%)	6,646 (15.01%)	80 (51.8; 0–758)	41 (34.2; 0–600)
Very preterm ≥28–31 weeks; *n* (%)	621 (1.40%)	68 (67.4; 0–498)	45 (57.30; 0–734)
Extremely preterm <28 weeks; *n* (%)	248 (0.56%)	42 (44.3; 0–341)	52 (61.4; 0–456)
Unknown term of birth	138 (0.32%)	94	45

* *The Mann-Whitney test did not show a significant difference between males vs. females and TREC and KREC values*.

Out of 44,287 samples, 321 (0.72%) were re-tested from the first DBS. Among the 321 re-tested samples, 168 had a low number of ACTB (<1,000 copies/μL) and, due to a lack of a sufficient amount of DNA in the sample, concomitant reduction of TRECs and KRECs were referred to as “inconclusive” and re-tested. The remaining 153 DBS with ACTB >1,000 copies/μL and TREC or KREC copy numbers below the respective cutoff values were considered “abnormal” and also re-tested. After re-testing, 256 newborns presented with normal results (TREC >6 copies/μL, KREC >4 copies/μL, ACTB >1,000 copies/μL); 65 neonates had positive results and required reevaluation. Out of this group, 11 (11/65; 16.92%) newborns had TREC value <6 copies/μL and 34 (34/66; 52.13%) retested children had KREC values <4 copies/μL. In two (2/65; 3.081%) newborns both TREC and KREC values were below the cut-off. In 18 (18/65; 27.70%) newborns, the poor-quality samples with undetected TREC and/or KREC copies and values of ACTB ≤ 1,000 copies/μL were detected, indicating insufficient efficacy of DNA extraction from DBS. In the case of eight newborns TREC and/or KREC copy values were below 1 copy/μL from the first blood samples (3 DBS punches). They were considered as urgent-positive and immediate admission to the hospital for clinical examination and flow cytometry was recommended, without waiting for the results from the second DBS. For the remaining 58 newborns, a second blood sample was requested. In the case of extremely and very preterm newborn (born <32 HBD) the second screening cards were taken when the child reached 32–34 weeks of gestational age. After re-testing the second DBS, one newborn had a positive result and was called to further immunological evaluation; 56 newborns had normal results (Table 2).

**Table 2:** Number of newborns, retests from the first DBS, and recalls in the study population.

	**Total**	**West pomerania szczecin**	**Mecklenburg-western pomerania greifswald**	**Brandenburg berlin**
Newborns	44,287	17,745	11,114	15,428
Re-tested sample from the1st DBS	321^*^	116	105	100
Re-call for confirmatory diagnosis after re-testing 1st DBS	7	3	2	2
Second sample	58	23	15	20
Re-call for confirmatory diagnosis after re-testing 2nd DBS	1	1	0	0

* *321 samples were re-tested from the first DBS (at least 2 punches): 168 “inconclusive”; results were related to low number of ACTB (<1,000 copies/μL) and subsequently reduced TREC and/or KREC values; - 153 “abnormal” results with ACTB >1,000 copies/μL had TREC and/or KREC copy numbers below the respective cutoff values*.

**Figure 3:**
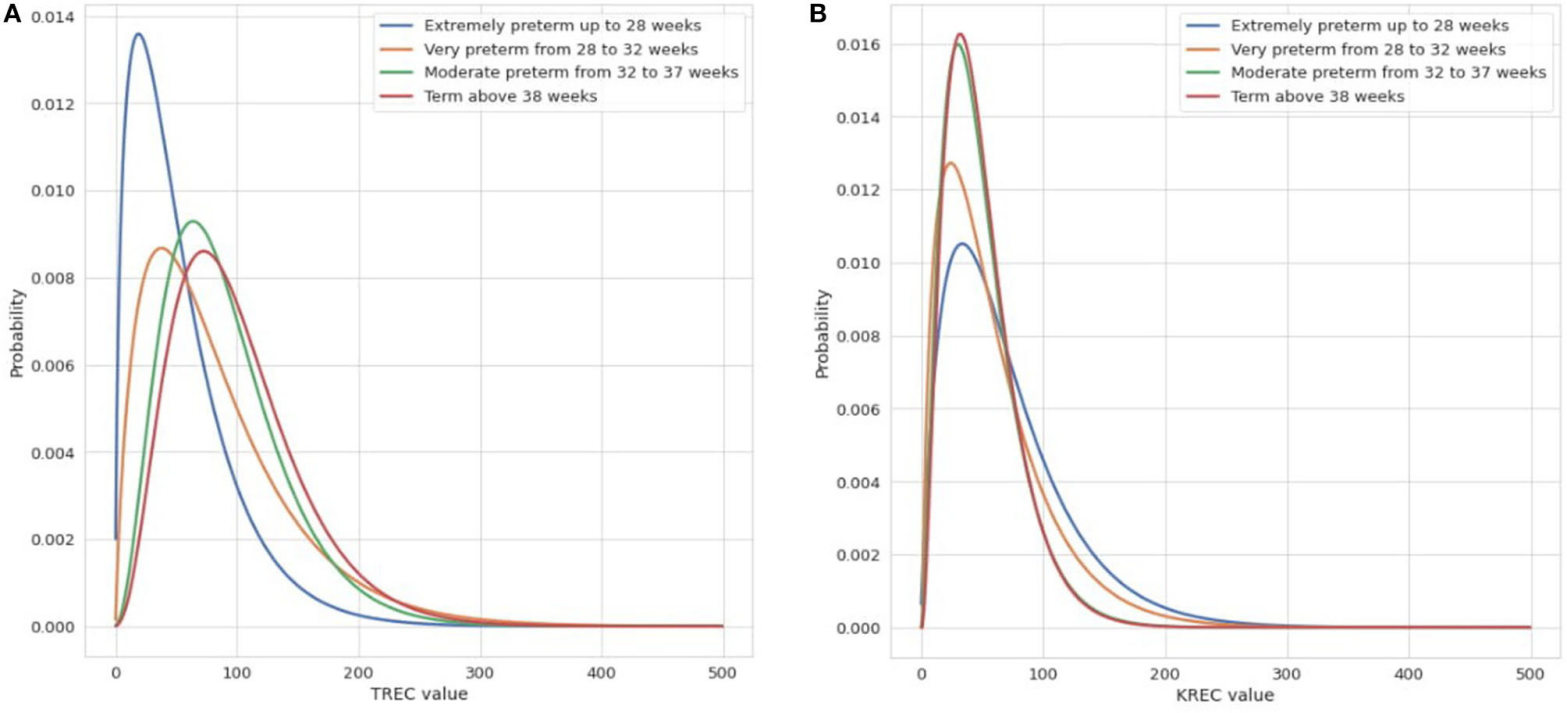
The distribution of TREC copies/μL **(A)** and KREC copies/μL **(B)** in groups divided by GA. The fitted beta distributions of TREC (copies/μL) and KREC (copies/μL) values in four study groups: extremely preterm (blue line), very preterm (orange line), moderate preterm (green line), term newborn (red line).

In the whole group of tested children, extremely premature newborns, born <28 weeks of gestation, had the lowest TREC values (median value−42 copies/μL) and the lowest spread of the results among the groups (Figure 3A). TREC values in 5% of the first DBS from this group were below the cut-off value of 6 copies/μL, however, they also tended to rise along with GA. In our study, the Kruskal Wallis test showed significant differences (*p* <0.05) in the values of TREC median between the studied groups of newborns divided according to the GA.

Regarding the KREC values, we did not notice higher rates of KREC results below cut off in children born <28 weeks of GA. However, in extremely premature newborns the spread of KRECs values was greater than in the other groups. Children born between 32 and 37 and >38 weeks of gestation had similar distributions of KREC values (respectively median value: 41 and 42 copies/μL). The Kruskal-Wallis test shows a significant difference (*p* <0.05) between the values of medians in the groups of newborns divided according to the GA (Figures 3A,B).

### Final Diagnosis

During the first 14 months of the “RareScreen” project, 44,287 newborns were screened for SCID. After testing the first screening cards, among 65 positive results with low TREC and/or KREC values, seven neonates treated as urgent-positive were recalled to the hospital for confirmatory tests. Examination of second screening samples from 58 newborns resulted in recalling to the hospital of one newborn. Overall, eight newborns were referred for further immunological evaluation. Confirmatory procedures (mostly lymphocyte subsets analysis) revealed one case of T-B^low^NK+ SCID with severe cartilage-hair hypoplasia (homozygous mutation in *RMRP* G.70A>G), one case of atypical T^low^B^low^NK+ CID without dysmorphic features and of unknown genetic defect, one case of autosomal recessive agammaglobulinemia, and one case of Nijmegen breakage syndrome. Among four other cases of T- and/or B-cell lymphopenia, one was related to the mother's immunosuppression during pregnancy and one to prematurity. The next one, a child with slight dysmorphic features, presented low TREC only, followed by persistent/repeatable T-cell lymphopenia in flow-cytometry with normal TCR repertoire and mitogen stimulation. However, due to persistent T-cell lymphopenia, a molecular test (NGS) was performed, revealing no underlying genetic defects. At the age of 7 months, T-cell lymphopenia almost resolved, however the child remains under observation. The last newborn was truly false positive with normal results of flow-cytometry. In children with SCID and CID (pt.1 and pt.2), higher proportions of TCR γ/δ expression and loss of naivety were observed. In patient 1, diminsihed lymphoproliferation to phytohemagglutinin was observed. In patient 4 with Nijmegen breakage syndrome, only standard karyotype was performed, which resulted in changes in chromosome 7 and 14 (characteristic for this disease).

Both children with SCID and CID underwent a successful HSCT at the age of 2 and 3.5 months of age, respectively. In the child with agammaglobulinemia, immunoglobulin replacement therapy (IgRT) was introduced. The child with Nijmegen breakage syndrome required two instances of IgRT and remains under regular immunological monitoring. The other children with transient T- and/or B-cell lymphopenia are doing well and the immunophenotyping results are either normal or at least definitely improved.

**Figure 4:**
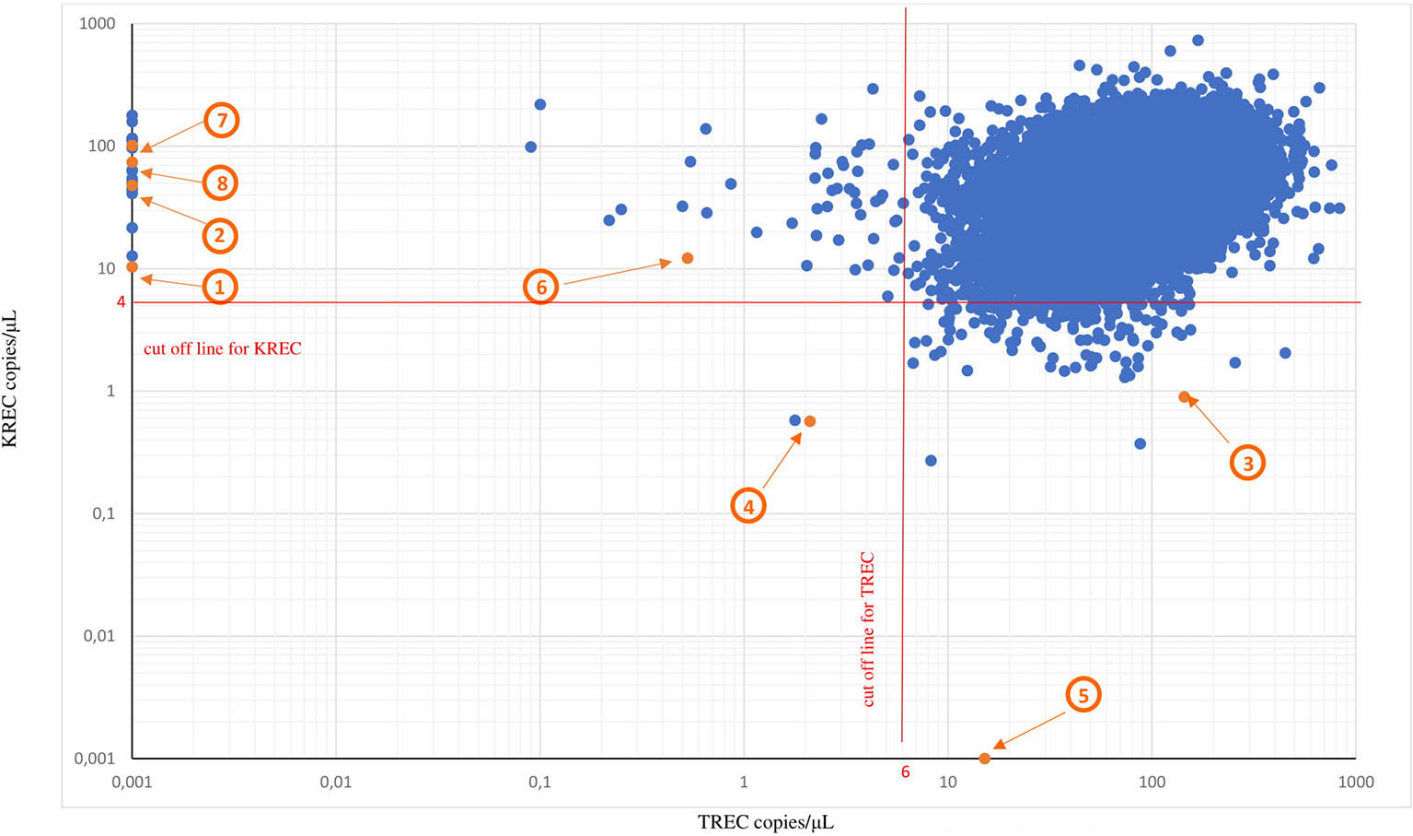
TREC and KREC copy numbers/μL from the first DBS and final diagnosis in eight newborns with severe PID. TREC and KREC copy numbers from the first DBSs.* Red lines represent cutoff values for TREC copies/μL (cut off value 6 copies/μL) and KREC (cut off value 4 copies/μL), respectively. Orange dots represent eight positive results from the first DBSs with T and/or B cell lymphopenia confirmed on flow cytometry. 1—T-B^low^NK+ SCID; 2—T^low^B^low^NK+ CID; 3—AR agammaglobulinemia; 4—Nijmegen breakage syndrome; 5—Transient B-cell lymphopenia due to mother's immunosuppression; 6—T- and B-cell lymphopenia due to prematurity; 7—Transient T- cell lymphopenia of unknown reason; 8—False positive of unknown reason. *The samples which had a low number of ACTB (<1,000 copies/μL) and concomitant reduction of TREC and KREC copy numbers were referred to as “inconclusive” because of a lack of DNA and samples were excluded from the graph.

The overall positive predictive value (PPV) was 50.0% (Table 3, Figure 4).

**Table 3:** Final diagnosis in eight newborns recalled for further immunological evaluation due to positive results in population newborn screening for severe PID.

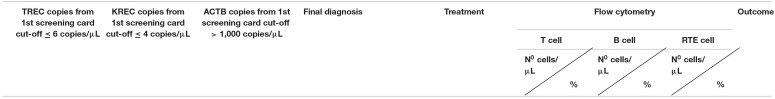
1	0–0	10.4–11.4	2,918–3,589	T-B^low^NK+ SCID Cartilage-hair hypoplasiaHomozygous mutation in *RMRP* g.70A>G	HSCT at the age of 2 months	70 (ref. 2,300–7,000)5 (ref. 60–85%)	130 (ref. 600–1,900) 9 (ref. 4–26%)	8.54 (ref. 49.78–79.60%)	Alive
2	0.0–0.0	48–88	3,020–8 895	T^low^B^low^NK+ CID Unknown genetic cause	HSCT at the age of 3.5 months	480 (ref. 2,300–7,000) 38 (ref. 60–85%)	240 (ref. 600–1,900) 18 (ref. 4–26%)	15.94 (ref. 49.78–79.60%)	Alive
3	144.5–163.4	0.9–0.6	6,186–10 409	AR agammaglobulinemia homozygous IGLL1-mutation *(IGLL1 c425C>T, p.Pro142Leu)* The parents are each heterozygous for this mutation	IgRT	4,170 (ref. 2,300–7,000) 93 (ref. 60–85%)	30 (ref. 600–1,900) 1 (ref. 4–26%)	80 (ref. 49.78–79.60%)	Alive
4.	2.1–3.1	0.6–0.7	4,104–4,356	Nijmegen breakage syndrome, homozygous deletion c.657_661del5 in *NBN* gene	IgRT	1 171 (ref. 1,700–3,600)56.06 (ref. 58–67%)	45 (ref 500–1,500) 2.17 (ref. 19–31%)	45.57 (ref. 50–74%)	Alive
						1,581^*^ (ref. 2,000–4,700)49.9^*^ (ref. 54.6–80.5%)	113^*^ (ref. 700–2,400) 3.57^*^ (ref. 10.0–30.7%)	22.7^*^ (ref. 50–74%)	
5.	12.1–22.1	0–0.2	4,909–3,208	Transient B-cell lymphopenia due to mother's immunosuppression during pregnancy (Mycophenolate Mofetil—in the first gestation weeks, prednisone, azathioprine, and tacrolimus)—flow cytometry results normalized with age	No	2,420 (ref. 1 700–3,600)95.61 (ref. 58–67%)	10 (ref 500–1,500) 0.38 (ref. 19–31%)	31.1 (ref. 50–74%)	Alive
						5,901^*^ (ref. 2,000–4,700)62.31^*^ (ref. 57.1–72.4%)	2,870^*^ (ref. 700–2,400) 30.3^*^ (ref. 18.4–37.5%)	56.6^*^ (ref. 52–57%)	
6.	0.5–0.1	12.18–7.0	755–1,117	T- and B-cell lymphopenia due to prematurity (GA−33 weeks). On molecular testing (WES)—no data for PID, in the *HBD* gene heterozygous pathogenic variant c.82G>T p. (Ala28Ser) was identified -the result is consistent with the genetic diagnosis of AD delta-thalassemia. Follow-up flow cytometry results improved with age	No	522 (ref. 1,700–3,600) 47.64 (ref. 58–67%)	222 (ref 500–1,500) 20.27 (ref. 19–31%)	45.57 (ref. 49.78–79.60%)	Alive
						1 133^*^ (ref. 2,000–4,700) 30.4^*^ (ref. 57.1–72.4%)	1 625^*^ (ref. 700–2,400) 43.59^*^ (ref. 18.4–37.5%)	27.1 (ref. 52–57%)	
						1 225^**^ (ref. 1,400–2,000) 39,77^**^ (ref. 66–76%)	813^**^ (ref 300–500) 26,41^**^ (ref. 12–22%)	29,57^**^ (ref. 55–77%)	
7	0.0–1.0	101.3–80.1	1,334–2,528	T- cell lymphopenia of unknown reason—follow-up flow cytometry results improved with age Unknown genetic cause	No	1,250 (ref. 1,700–3,600) 56.14 (ref. 58–67%)	635 (ref. 500–1,500) 2.,53 (ref. 19–31%)	–	Alive
						637^*^ (ref. 2,000–4,700) 37.7^*^ (ref. 57.1–72.4%)	904^*^ (ref. 700–2,400) 53.47^*^ (ref. 18.4–37.5%)	56.1^*^ (ref. 52–77%)	
						2,745^**^ (ref. 2,800–5,700) 50.6^**^ (ref. 58.5–77.1%)	2,240^**^ (ref. 700–2,800) 41.3^**^ (ref. 15.7–34.1%)	62.6^**^ (ref. 55–77%)	
8	0.0–0.0	74.3–54.8	1 639–705	False positive	No	2,880 (ref. 2,300–7,000) 61 (ref. 60–85%)	940 (ref. 600–1,900) 20 (ref. 4–26%)	68.87 (ref. 60.9–80.6%)	Alive

*Range of results from the first screening card includes 3 measurements (first test and repetition in duplicate)*.

*Differences in age-related values of flow-cytometry parameters arise from differences in laboratory specific referential ranges of flow-cytometry*.

*SCID, severe combined immunodeficiency; CID, combined immunodeficiency; TREC, T-cell receptor excision circles; KREC, kappa-deleting element recombination circle; ACTB, beta-actin; HSCT, hematopoietic stem cell transplantation; AR, autosomal recessive trait of inheritance; IgRT, immunoglobulin replacement therapy; GA, gestational age; PID, primary immunodeficiency diseases; RTE, recent thymic emigrants; AD, autosomal dominant trait of inheritance; WES, whole exome sequencing; GA, gestation age*.

* *repeated flow cytometry in 2nd-3rd month of life*.

** *repeated flow cytometry in 7nd-8th month of life*.

## Discussion

Six decades have passed since Robert Guthrie developed the population newborn screening test for PKU (1). During this time, the NBS evolved from detection of phenylalanine levels on filter paper to application of DNA-based technics for early identification of a number of rare genetic disorders.

Many authors agree that SCID, and other severe forms of PID, are an important health problem with a known natural history and available treatment, which meets the Wilson and Junger criteria (18, 19).

Since 2008, TREC analysis has been used as a screening method for severe forms of primary T-cell lymphopenia (2, 5, 20, 33). Further research enabled the extension of the qPCR method to simultaneous measurement of TREC and KREC values. The method, which includes KREC analysis to assess potential B-cell lymphopenia, allows for additional identification of patients with XLA, Nijmegen breakage syndrome, and purine nucleoside phosphorylase (PNP) deficiency (9, 34). However, TREC screening will not identify infants with SCID in which a molecular defect lies downstream of T-cell receptor rearrangement, including variants in *ZAP70*, MHC class II (major histocompatibility complex class II), and ADA (delayed-onset disease) (35–39).

Infants with SCID and other forms of PID are susceptible to life-threatening infections of different origins, as well as secondary infections induced by life vaccines (40). Early diagnosis was made possible by testing T- and/or B-cell lymphopenia with NBS procedures, thereby significantly improving the life of affected children. Detection of PID in the first weeks of life enables an effective treatment with HSCT, enzyme replacement therapy, gene therapy, and/or immunoglobulin replacement therapy (16, 17, 19).

Nowadays, more and more countries have already introduced or are considering the population NBS for PID using the evaluation of TREC alone or in combination with KREC analysis (19, 41).

In our study we used the combined TREC and KREC assay. It is similar to the methods used in other centers, including those in Sweden, Iran, and Spain (33, 34, 42, 43). The study by Borte et al. gave the first specific TREC/KREC cut-off values for SCID NBS after retesting DBS samples of known SCID patients. Their findings were supported by further studies which then allowed conclusions about specificity and sensitivity (34). Barbaro et al., performed a population wide NBS using a combined TREC/KREC test with a cohort study size of 58.834 Swedish newborns from Stockholm county (33). Their findings were supported by studies performed in Sweden, Spain, and Germany with a specificity of >99% and sensitivity of >95% for the detection of severe T- and /or B-cell lymphopenia in dry blood material (24, 34). The study from the Stockholm/Uppsala and Leipzig regions showed a PPV of 46% for severe T- and/or B-cell deficiency using the threshold values <6 copies/μL for TREC and <4 copies/μL for KREC. After evaluating the control-screening card, a PPV of 100% was found (34). In comparison, the following PPVs for severe T-cell deficiency was published based on data from the USA for TREC screening alone: State of Wisconsin 47%, State of California 31%, State of New York 18%, and State of Massachusetts 37%. Currently, the negative predictive value is 99% (44).

During the first 14 months of the RareScreen project, 44,287 newborns from Poland and Germany were screened. Overall, nine positive cases (including seven urgent-positive) were referred for immunological assessment starting with flow cytometry and followed by further investigations. Approximately, 1:5,536 of tested newborns underwent further confirmatory procedures in our cohort, while in the United States the number ranged from 1:735 to 1:7,500 depending on the state, and in Sweden it was 1:20,000 (21, 33).

Out of eight newborns, one case of SCID and one case of CID were diagnosed. The recognition of two patients with SCID/CID in the studied population of 44,287 newborns demonstrates the efficacy of NBS with TREC evaluation in early detection of affected individuals, but at the same time it requires careful consideration as the number of screened newborns is still very limited. The next child, presenting with normal TREC but low KREC levels, was diagnosed with autosomal recessive agammaglobulinemia. In another newborn, with low KREC and diminished TREC as well as microcephaly and dysmorphic features, Nijmegen breakage syndrome was recognized with a variant in the *NBN* gene typical for the Slavic population. These two cases prove that the combined TREC/KREC testing should be considered as a powerful tool for initial screening of not only SCID suspected patients but also individuals with other severe PID. The introduction of combined TREC and KREC tests can significantly advance early diagnosis of inborn errors of immunity, such as agammaglobulinemia, Nijmegen breakage syndrome, ataxia-teleangiectasia or DiGeorge syndrome, as well as late-onset ADA SCID (33, 45–48). It allows for the early introduction of the proper treatment and prophylaxis (ex. HSCT, IgRT) before infectious complications appear or to avoid X-ray exposition in the case of Nijmegen breakage syndrome, ataxia-teleangiectasia, or other radiosensitive syndromes.

One positive result with a low KREC value was related to the mother's immunosuppression during pregnancy. The mother was treated with Mycophenolate Mofetil (in the first weeks of gestation), prednisone, azathioprine, and tacrolimus after a liver transplant due to autoimmune hepatitis. It is known that azathioprine may cross the placenta and B lymphocytes are more sensitive to drug-induced apoptosis than T lymphocytes (49). Two similar cases were described by Filipe et al. in Spain (24). Although the patient's initial flow cytometry on the eleventh day of life revealed significant lymphopenia, including reduced numbers of CD19+B cells and milder diminished number of CD4+/CD3+ T cells and NK cells, repeated evaluation at the first and second month of age showed a normalisation of lymphocytes. This case shows that communication between neonatal/paediatric departments and screening laboratories is crucial in successful patient management. The knowledge about what medical treatment mother and/or the newborn underwent, may help to determine adequate follow-up steps.

In one premature neonate, born at the thirty-third week of gestation, initial low TREC resulted in recall for further evaluation. Although the first lymphocyte immunophenotyping revealed reduced numbers of CD19+B cells, CD3+, and CD4+naïve T cells as well as reduced T cell subpopulations, normalisation of CD19+B and significant improvement of CD3 subpopulation were noted in the repeated tests at the second and seventh month of life.It is worth underlining that TREC levels obtained in our study decreased with lower GA. It was especially apparent in children born <28 weeks of gestation. Such observations have been reported in several other publications (32, 50). Regarding the KREC values, we did not notice a higher rate of KREC results below cut off in children born <28 weeks of GA. However, a significant difference between KREC values in the groups of newborns divided according to the GA was shown. In groups of extremely premature and very preterm newborns, the spread of KREC values was greater than in children born ≤ 32 weeks. In a study by Kanegae et al., KREC values did not vary according to GA and in the studies by Barbaro et al. and de Felipe et al. KREC levels did show a downward trend with decreasing GA (25, 50). Further evaluation is needed and may provide data to define the reason for these differences and their clinical implications.

In the whole study group, there was one truly false positive case (low TREC with normal T-cell in flow-cytometry) of unknown reason. Pilot screening performed by Holodnij et al. proved that blood collection is crucial for proper determination of TREC and KREC values. Newborn heel blood should be applied directly to the screening card. Blood applied from capillaries or heparinized blood collection tubes should be avoided as heparin is known to inhibit DNA polymerases resulting in low values of TREC and KREC and/or ACTB copies number (51). Furthermore, insufficiently soaked screening cards also tend to lead to low TREC/KREC results. The company providing SCID kits recommend using the center part of DBS, which could not be ensured for all used samples, especially in the beginning of our study.

In our study many assays, depending on necessity and availability, were used to confirm the diagnosis of severe PID following positive screening tests. The confirmation algorithms vary in different countries or even by state. In some centers they are routinely limited to complete blood count (CBC) and lymphocyte profile only with extension to TCR repertoire, mitogen stimulation, or, if needed, to maternally engrafted cells assessment (32, 52, 53). Rechavi et al. suggested an initial confirmatory panel with complete blood count, full lymphocyte profile, and TREC quantification in the peripheral blood (32). In our region CBC, lymphocyte subset with TCR, and mitogen tests are done routinely, followed by supplementary assays according to clinical and laboratory deviation.

In summary, we can conclude that newborn screening programs, including TREC and KREC, followed by detailed immunological assessment, are of great value to avoid complications in children with undiagnosed PID.

## Strengths and Limitations of the Study

The strength of our study is the introduction of a combined TREC and KREC analysis, which allows the detection of not only SCID but also other severe PID with accompanying T- or B-cell lymphopenia. The recall frequency of 1: 5,536 is acceptable, however, we can expect that with growing experience (at the departments of neonatology and in NBS laboratories) and populations screened the frequency of recalls will be further reduced. Positive predictive values for SCID and other severe PID was 50.0% which was comparable to other studies (45).

As from August 2019, the national NBS program based on TREC has only been implemented in Germany and the comparison of pro and cons between both programs (with or without KREC) will be possible. Based on the literature data and our still very limited experiences, we believe that by determining KREC, patients with congenital severe B-cell lymphopenia can also be identified. With the help of KREC copies evaluation, compared to an isolated TREC screening, SCID patients who suffer from a prevalent B-cell deficiency at birth, such as the “delayed-onset” ADA or purine nucleoside phosphorylase (PNP) deficiency, can also be detected (9, 34, 39). In addition, using KREC screening newborns with agammaglobulinemia (e.g., Bruton disease) can be identified at birth and consequently treated before serious infections occur. This leads to the prevention of lethal and serious infections with irreversible organ damage (mostly bronchiectasis), which has a lasting impact on the development of the affected children (54). A combined TREC and KREC screening is also advisable in terms of the fact that it would not involve any technically complex changeover, however the established screening algorithm would need to be changed.

However, the small population of selected regions is a limitation of the study, not allowing conclusions on the exact prevalence and incidence of SCID and other severe PID in other countries. At the same time, the study shows that cross-border cooperation between several screening centers can very efficiently combine their complementary expertise on such rare diseases. This can compensate for the fact that corresponding capacities cannot be maintained at all locations.

This is the first population screening study that allowed identification of newborns with T and/or B immunodeficiency in Central and Eastern Europe. It shows that newborn screening for severe PID, including different types of SCID, is effective and should be implemented into population obligatory panels for NBS and, to this effect, international cooperation proved to be efficient.

## Data Availability Statement

The datasets generated for this study are available on request from the corresponding author.

## Ethics Statement

The studies involving human participants were reviewed and approved by Ethic Committee at the Pomeranian Medical University in Szczecin, Poland (EA2/119/18), Ethic Committee of the University Medicine Greifswald, Germany (BB 081/18), Ethic Committee of the University Medicine Charité, Berlin, Germany (EA2/119/18). Written informed consent to participate in this study was provided by the participants' legal guardian/next of kin.

## Author Contributions

MG and MP designed the concept of the manuscript. TW, MG, MN, MO, MP, SS, OB, and TW designed the concept of the RareScreen project. MG, TW, and SS were coordinators for the project funding. TW, KD, OB, JK, EB, and NS did the laboratory work. KD and MO did the statistical analysis. MG, MP, KD, TW, IO, OB, and MFP analysed and interpreted the data. KD drew figures and summarized the tables. MG, MP, KD, IO, HR, EK-Z, MFP, BB, EAB, HvB, CM, and MW collected the relevant information and references. MG, MP, KD, TW, IO, and MO wrote the manuscript with contribution from all co-authors. All authors read, critically reviewed, and approved the final version of the manuscript.

## Conflict of Interest

The authors declare that the research was conducted in the absence of any commercial or financial relationships that could be construed as a potential conflict of interest.
